# Synthesis of Hybrid Tin‐Based Perovskite Microcrystals for LED Applications

**DOI:** 10.1002/advs.202403835

**Published:** 2024-07-08

**Authors:** Jesus Sanchez‐Diaz, Jhonatan Rodriguez‐Pereira, Samrat Das Adhikari, Iván Mora‐Seró

**Affiliations:** ^1^ Institute of Advanced Materials (INAM) Universitat Jaume I. Av. de Vicent Sos Baynat Castellón de la Plana 12006 Spain; ^2^ Center of Materials and Nanotechnologies, Faculty of Chemical Technology University of Pardubice Nam. Cs. Legii 565 Pardubice 53002 Czech Republic; ^3^ Central European Institute of Technology Brno University of Technology Purkynova 123 Brno 61200 Czech Republic

**Keywords:** LED, microcrystalline powder, Pb‐free perovskites, precursor synthesis, tin perovskites

## Abstract

Considerable focus on tin‐based perovskites lies on substitution to leadhalide perovskites for the fabrication of eco‐friendly optoelectronic devices. The major concern related to tin‐based perovskite devices are mainly the stability and the efficiency. However, thinking on the final commercialization scope, other considerations such as precursor stability and cost are major factors to carry about. In this regard, this work presents a robust and facile synthesis of 2D A_2_SnX_4_ (A = 4‐fluorophenethylammonium(4‐FPEA); X = I, Br, I/Br) and 3D FASnI_3_ perovskite microcrystals following a developed synthesis strategy with low‐cost starting materials. In this developed methodology, acetic acid is used as a solvent, which helps to protect from water by making a hydrophobic network over the perovskite surface, and hence provides sufficient ambient and long‐term inert atmosphere stability of the microcrystals. Further, the microcrystals are recrystallized in thin films for LED application, allowing the fabrication of orange, near‐infrared and purered emitting LEDs. The two‐step recrystallized devices show better performance and stability in comparison to the reference devices made by using commercial precursors. Importantly, the developed synthesis methodology is defined as a generic method for the preparation of varieties of hybrid tin‐based perovskites microcrystals and application in optoelectronic devices.

## Graphical abstract

A novel synthesis strategy has produced 2D hybrid tin halide perovskite microcrystals, featuring cost‐effectiveness and scalability. These microcrystals exhibit ambient stability and endure long‐term storage in inert atmospheres. Utilized as perovskite precursors, they enable the fabrication of Pb‐free, eco‐friendly pure‐red emitting LEDs. Recrystallized samples demonstrate superior performance compared to conventional methods, highlighting their remarkable attributes.

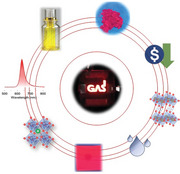

## 1 Introduction

During the past decade, Pb‐based halide perovskite light‐emitting diodes (PeLEDs) external quantum efficiency (EQE) has jumped from values <1% in 2014^[^ ^1^ ^]^ to 8.53% in 2015^[^ ^2^ ^]^ and recently has reached 28.9% reported by Tae Woo Lee and coworkers.^[^ ^3^ ^]^ Research and developments of PeLEDs is under progression toward the commercialization considering mainly the following criteria: i) high performance and stability, ii) low‐temperature synthesis and large‐scale producibility, iii) use of non‐critical raw‐materials, iv) vast tunability of visible color emission.^[^ ^4^ ^]^ The relatively low long‐term stability of the PeLEDs is perceived for the 3D perovskites with ABX_3_ structure and arises from the degradation under ambient conditions anticipated from their low formation energy. The introduction of bulky ammonium cations (L) into the 3D perovskites leads to a reduced 2D dimensionality, classified as Ruddlesden‐Popper (L_n+1_A_n‐1_B_n_X_3n+1_) or Dion‐Jacobson (L_n_A_n‐1_B_n_X_3n+1_) perovskite based on their structural reorganization, these classes of perovskites are known as 2D layered perovskites, where L is a bulky cation, known as spacer cation.^[^ ^5^, ^6^ ^]^ 2D halide perovskites are in the area of interest because of their flexibility of changing the organic cation to have tunable structural and optoelectronic properties on top of an improved chemical and optical stabilities, increasing exciton binding energy.^[^ ^7^ ^]^

Noteworthy, the commercialization of the best‐performing halide perovskite‐based optoelectronic devices faces the drawback of containing hazardous Pb.^[^ ^8^ ^]^ Therefore, the present scenario of the PeLEDs research and development is mostly looking forward to the Lead‐free halide perovskites (LFP).^[^ ^9^, ^10^ ^]^ In this sense, a set of Pb‐free double perovskites, foreign metal ion(s) doped vacancy‐ordered double perovskites, 2D perovskites are reported providing highly intense photoemission.^[^ ^11^ ^]^ However, the origin of the photoemission of most of these systems is from self‐trapped excitonic (STE) emission, which is characterized by: i) wide bandgaps, ii) large stokes shift, and iii) wide bandwidth photoemission features.^[^ ^12^ ^]^ Nevertheless, PeLEDs from STE emitting Pb‐free halide perovskites are still elusive to have electroluminescence (EL) from those visible‐light emitting LFPs or presenting low EQE.^[^ ^13^ ^]^ Despite the photoluminescence quantum yield (PLQY) efficiencies of many STE emitting LFPs are very promising and show over 50% and even more, there are a few reports exhibiting EL, and the as‐made device performances are considerably low, considering low external quantum efficiencies (EQE), low luminance, etc.^[^ ^13^, ^14^ ^]^ In this regard, PeLEDs made of band‐to‐band emitting perovskites provide electroluminescence with the traditional device structure, and even with lower PLQYs (≈2%) compared to the ones with STE, show higher EQE for PeLEDs.^[^ ^15^ ^]^ Therefore, Pb‐free band‐to‐band emitting perovskites are a valuable alternative for PeLEDs in order to address the toxicity of Pb.

Tin iodide perovskites have been proven to have their EL from the band‐to‐band emission,^[^ ^15^, ^16^ ^]^ in contrast with bromide perovskites exhibiting in most of the cases STE emission. One of the promising Pb‐free perovskites for PeLEDs applications is phenethylammonium tin iodide (PSI), a 2D layered perovskite, that consist of individual layers of corner‐shared tin iodide octahedra separated by repetitive organic spacer cations (herein, phenethylammonium) that importantly emits in the pure red window (≈630 nm). Normally, the active layer deposition is followed by the traditional procedure, where SnI_2_ and phenethylammonium iodide is dissolved in DMF/DMSO and a certain concentration of the solution is spin‐coated followed by the annealing to crystallize their films. However, the developed methods are not cost‐effective for the deposition of thin‐film active layers.

It is well‐established that using perovskite as a precursor, rather than commercial salt precursors, is advantageous as it results in better thin‐film quality, reduced surface defects, improved surface coverage, and enhanced stability. However, the reported synthesis strategy for tin iodide perovskite microcrystals involves a high quantity of HI, which leaves residual water in the microcrystals, leading to further oxidation of Sn^2^⁺ to Sn⁴⁺. Therefore, a robust and generic synthesis method that provides ambient stability and scalability is yet to be developed. In summary, the challenge to commercialize tin‐based PeLEDs lies on the following points: i) low device performance and luminance, ii) easy oxidation of Sn^2+^ into Sn^4+^, and iii) cost‐effective fabrication, which compromises their stability. In this work, we have developed a low‐cost synthesis strategy for the fabrication of Sn‐based perovskite microcrystals using low‐cost precursors such as tin oxide (SnO) as tin source and acetic acid as solvent. We show that this methodology significantly reduces precursor cost at the same time that increases precursor stability. This is the first report of ambient‐stable tin iodide microcrystals as perovskite precursor. We discovered that acetic acid acts as ligand to protect from Sn^2+^ and I^−^ oxidation and hence contributes to the stability of the microcrystals. Furthermore, the microcrystals are redissolved in a solvent mixture of DMF:DMSO for the deposition of perovskite thin films and their application for PeLEDs. We have extended this synthesis to other halide perovskites as 4‐fluorophenethylammonium tin iodide halide perovskite (4FPSI) for which we have obtained PeLEDs presenting higher performance than for PSI‐based perovskites, showing the generality of the methodology. Beyond the stability and reduced precursor cost, this methodology provides better device performance and stability compared to the reference devices prepared with commercial precursors.

## 2 Results and Discussion

In the advanced synthesis methodology here reported, acetic acid serves as the solvent, playing a pivotal role in maintaining stability, as follows: i) Partially ionized acetate ions coordinate with Sn^2+^ at the perovskite surface, and hence passivating Sn^2+^. ii) Protonated acetic acid engages in hydrogen bonding with its polar hydroxyl groups and iodide ions, safeguarding iodides from oxidation. iii) The hydrophobic nature of acetic acid aids in purifying microcrystals in non‐polar solvents like hexane, facilitating the removal of unreacted precursors and solvents while preventing water from interacting with the perovskite surface. The collective multifunctionality of acetic acid ensures the robust and scalable synthesis of high‐purity perovskite crystals with excellent stability, even in ambient conditions for weeks. Additionally, these microcrystals can be stored in an inert atmosphere (N_2_) for months to a year for future use.

Traditional solution‐processed synthesis of tin iodide perovskite microcrystals is carried out in excess hydroiodic acid (HI), where HI has two‐fold functionalities: the protons accelerate the reaction to produce single‐crystalline microcrystals, and the iodides are anion source.^[^ ^17^ ^]^ However, HI consists of an excess amount of water, and an overabundance amount of iodide beyond the stoichiometry, which dictates the perovskites to degrade. The presence excess of iodide readily oxidizes to iodine, and the iodine adsorbs over the surface of the microcrystal powder and degrades the perovskite further.^[^ ^18^ ^]^ In addition, water is necessary for the washing of perovskite microcrystals to remove excess HI, which is challenging to dry before the degradation, therefore, it cannot be stored inside the glovebox for further processing of device fabrication. To overcome these challenges, we used acetic acid as solvent, and a minimum quantity to maintain stoichiometry of HI was used as iodide source. The drawback with washing of the perovskite microcrystals have been also solved with the use of acetic acid as solvent, which has hydrophobic ending methyl‐group and this helps with the purification of the microcrystals using non‐polar solvents which do not interfere with the perovskites.

**Figure 1:**
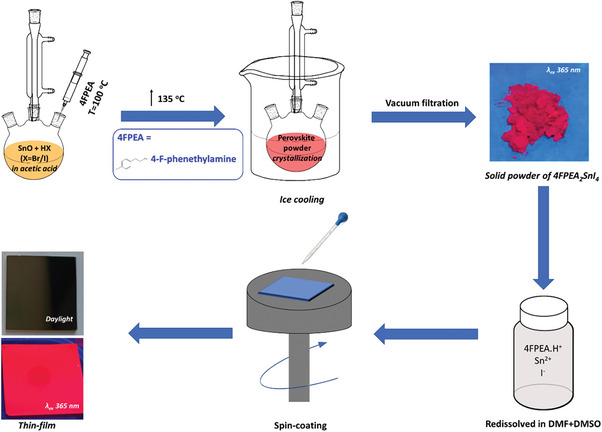
Schematic presentation of two‐step recrystallization of 4FPSI perovskites: synthesis of microcrystal powder, and its thin‐film deposition.

In particular, 4‐fluorophenethylammonium tin iodide halide perovskite (4FPSI) microcrystals have been synthesized using SnO, and HI as metal and halide precursors, along with H_3_PO_2_ to stabilize HI in acetic acid as solvent, followed by the addition of 4‐fluorophenethylamine as spacer cation. The as‐prepared 4FPSI perovskite powders were washed thoroughly in hexane to remove excess iodine. A detailed synthesis procedure is provided in supporting information, and the reaction scheme is provided in **Figure** **1**. After the synthesis of 4FPSI microcrystals, the powder is dissolved in 4:1 DMF/DMSO, and spin‐coated to recrystallize as thin‐film. Our developed synthesis methodology has proven to provide high‐quality, long‐term stable, and phase stable red‐emitting 2D tin iodide perovskite powder, which is used as the precursor for 2D PeLED emitter layer. While the major aspect of this work is the preparation of low‐cost and stable perovskite microcrystals, the benchmark of this work is the use of those microcrystals for the application on PeLEDs.

We investigated also the preparation of phenethylammonium tin iodide (PSI) microcrystals. Following the traditional approach (without acetic acid),^[^ ^17^ ^]^ PSI microcrystal powder was prepared following wet‐chemical method, however, we have experienced quick oxidation of Sn^2+^ to Sn^4+^ and iodine adsorption into the halide perovskite powder, which further degraded the perovskite powder to form multiple side products. Alongside, the as‐prepared PSI microcrystals showed quick phase transformation to the self‐trapped emitting phase.^[^ ^19^ ^]^ Herein, the phase transformation and the degradation take place simultaneously. Following our modified approach, the as‐prepared PSI powder is stable for at least 3 days as per the laboratory reports under ambient atmosphere and shows great stability of >6 months under inert atmosphere and could be stored for mentioned stability period to use as perovskite precursor for the preparation of thin‐film devices. The X‐ray diffraction (XRD) and optical characteristics of PSI microcrystals powder obtained with the new method here reported is provided in Figure S1a–c (Supporting Information). A photograph of degraded PSI sample prepared following traditional synthesis procedure and its XRD pattern is provided in Figure S2a (Supporting Information). XRD of this sample, see Figure S2b (Supporting Information), confirms the impurity peaks from degraded perovskite, and the comparison between Figures S1 and S2 (Supporting Information) evidence the suitability of our modified synthesis methodology. An intense orange emission is observed from the degraded PSI powder, see Figure S2b (Supporting Information), originated from the phase transformed product, which provides self‐trapped emission unsuitable for efficient LED applications.

The phase transformation has been justified with the support of recent literature report on octylammonium tin iodide, where two different phase of the said material was formed under different synthetic condition due to the Sn‐concentration into the materials. In our case, we have synthesized PSI microcrystals following colloidal approach (details provided in supporting information), where the XRD pattern has a repetitive peak of 4.68° (see Figure S3a, Supporting Information) and it has a bright orange broadband emission originated from STE (evidenced from large stokes shift) centered at 662 nm (see the PL and PLE spectra provided in Figure S3b, Supporting Information), while the microcrystals synthesized following our modified approach provides band‐to‐band narrowband emission with a small stokes shift, and its XRD pattern shows a different value of repetitive peak interval of 5.4°. From this observation, we anticipated that the different peak patterns are due to two different phases of PSI. The XRD patterns of degraded PSI contains some extra peaks due to the presence of degraded impurities and also contains the assigned peaks from the phase transformation, comparison provided in Figure S3c (Supporting Information). Interestingly, following the traditional synthesis approach, when 4‐fluoro phenethylamine was substituted with phenethylamine, an extended improvement in optical stability was observed, and the phase transformation was absent. Figure S4a–b (Supporting Information) provides the comparison between 4FPSI and PSI under varied excitation wavelength showing 4FPSI possess red emission originated from free excitons, while PSI have a wider phase‐transformed orange emission originated from STE. This improved optical stability of 4FPSI is anticipated from the electrostatic interaction between the fluorine groups at para‐position of phenethylamine.^[^ ^20^ ^]^ Taking into account the stability improvement on changing PEA to 4FPEA, we have chosen 4FPSI as our focused material for PeLEDs device fabrication. Despite the improved optical stability of 4FPSI synthesized following the traditional procedure, it has some limitations:

i) Use of large amounts of HI contains considerably large amount of water, which promotes degradation of perovskites (Sn^2+^ oxidation, hydrolysis of Sn^2+^, oxidation of I^−^ to I_2_, etc.) and hence hampers the stability of the microcrystals.
ii) The microcrystals are difficult to dry (purify) when they contain a large quantity of HI. To remove the excess HI+H_2_O where the microcrystals are dispersed, water is needed, which can even degrade the perovskite during/after washing.
iii) If the microcrystals are not purified, they cannot be implemented for device fabrication or even other application.

Keeping in mind all these points, using acetic acid as solvent has some benefits on washing the as‐prepared microcrystal powders during the purification process. As mentioned before, during the purification of tin iodide perovskite microcrystals in ambient, iodide oxidizes inevitably along with the Sn^2+^ oxidation. Acetate ions from acetic acid coordinates with Sn^2+^, while the pronated acetic acid form hydrogen bonding with the iodide ions, thus creates a hydrophobic network over the perovskite.^[^ ^21^ ^]^ Water adversely affects the stability of Sn^2^⁺ ions; hence, hexane was employed as the washing solvent due to its ability to form a hydrophobic network on the surface. In this context, washing with hexane does not interfere with Sn^2^⁺, unlike water, and effectively removes unreacted acetic acid from the system. Additionally, hexane offers an advantage in the purification process by facilitating the removal of iodide‐oxidized products, thereby aiding in the purification of the as‐prepared material. To justify this, as‐prepared unwashed PSI sample was kept for 2 h, then the sample was washed for three times in hexane, and the washed hexane contained some iodide oxidized product (mainly, I_3_^−^), characterized by UV–vis absorption spectra presented in Figure S5 (Supporting Information). The absorption peak of I_3_^−^ was significantly quenched after three rounds of washing. However, no iodide oxidized products were obtained for 4FPSI microcrystals, ensuring the complete reaction of HI as iodide the source. Alongside, acetic acid helps to keep away water during the synthesis, and hence facilitates the formation of high‐quality product.

**Figure 2:**
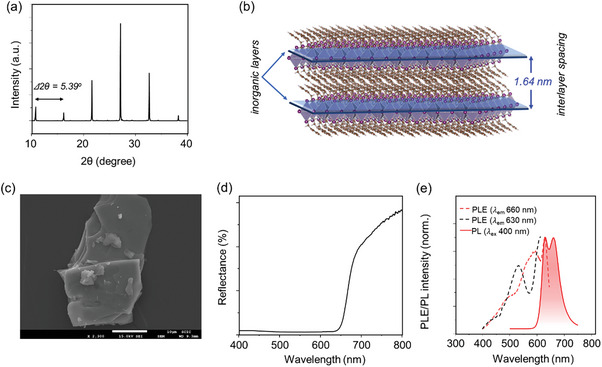
a) XRD pattern of 4FPSI microcrystal powder. b) Atomic model of 4FPSI perovskite structure, and their interlayer distance. c) Scanning electron microscopy (SEM) image, d) diffused reflectance spectrum, and e) PL/PLE spectra of 4FPSI microcrystals. The scale bar in (c) is 10 µm.

The characterizations of 4FPSI perovskite microcrystal are presented in **Figure** **2**. Figure 2a presents the X‐ray diffraction (XRD) pattern of as prepared 4FPSI microcrystals, showing repetitive XRD peaks with the interval of ≈5.39°, which corresponds to the interlayer distance of the inorganic layers ≈1.64 nm calculated using Bragg's equation (see Supporting Information), as shown in Figure 2b.^[^ ^22^ ^]^ Figure 2c presents the scanning electron microscope (SEM) image of the 4FPSI microcrystals, revealing the crystalline nature of the product. The diffused reflectance spectra of the as‐prepared microcrystals are shown in Figure 2d, shows the characteristics of direct bandgap semiconductor with a bandgap of 1.9 eV, calculated using Tauc plot, see Figure S6 (Supporting Information). Photoluminescence (PL) and PL excitation (PLE) spectra are presented in Figure 2e, which shows a small stokes shift hence confirms the emission originates from the band‐to‐band emission. The dual peak PL observed is due to the exceptional crystalline structure found in the 2D halide perovskite microcrystals, which has been observed for 2D lead halide perovskite as well. In a report by Nag and co‐workers,^[^ ^6^ ^]^ spatially resolved PL of 2D BA_2_PbI_4_ perovskite microcrystals show the evolution of longer wavelength peak was from the edges of the microcrystals, while the shorter wavelength peak was from the isolated lead halide layers. This observation is not limited to the 2D lead halide perovskites only, but also has been reported in case of 2D BA_2_SnI_4_ perovskite as well, hence this optical characteristic is unique for the pure 2D single crystalline perovskites.^[^ ^17^ ^]^ In our case of 4FPSI microcrystals, the higher‐energy band centered at 629 nm is attributed to the electronic state of the isolated Sn‐I layers, while the lower‐energy band centered at 659 nm arises from the perovskite microcrystals' edges, and hence the lower‐energy emission depends on the thickness of the perovskites.^[^ ^17^ ^]^ Notably, both the PL peaks are excitonic in nature, and their time resolved PL (TRPL) studies revealed decay lifetime of 4.96 ns (λ_em_ 629 nm), and 5.1 ns (λ_em_ 659 nm), respectively, see Figure S7 (Supporting Information). The shorter lifetime value justifies both the emissions are excitonic in nature. The obtained PLQY of 4FPSI microcrystals was 1.23%.

Moreover, the developed synthesis methodology is highly scalable, where the amount of the twenty‐time scale‐up product yield was also in the same range to the batch synthesis, provided in supporting information, and the characterization of the scalable product is provided in Figure S8 (Supporting Information). In addition, the product was stable for >7 days under ambient condition, and more than a year inside the glove box, characterizations are provided in Figure S9 (Supporting Information).

**Figure 3:**
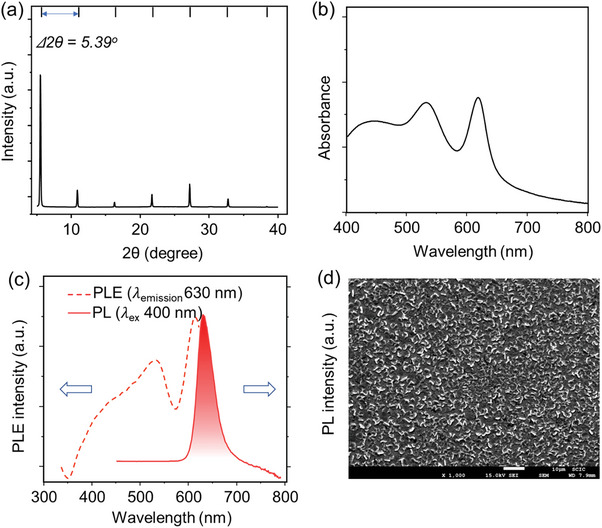
a) XRD pattern, b) UV–vis absorbance, c) PL/PLE, and d) top‐view SEM image of 4FPSI thin film prepared following the recrystallization of the microcrystals.

Thin films were prepared following two‐step recrystallization strategy, where the perovskite microcrystals were further dissolved in a mixture of DMF and DMSO (4/1 V/V) followed by filtering the solution with a 0.45 µm hydrophilic filter, and spin‐coated onto a glass substrate. The as‐prepared thin films are further characterized, see **Figure** **3**. Figure 3a presents the XRD pattern of 4FPSI thin film which matches the peaks from the prepared microcrystals powder, confirming high crystallinity of our films. Figure 3b,c presents the UV–vis absorption spectrum, and corresponding PL/PLE spectra of the thin film, respectively. Note that for thin films just a single PL peak is observed, compare Figures 2e and 3c, as the thin film lacks the presence of the lower‐energy band. As explained in the previous section, lower‐energy band originates from the edges of the perovskite layer, and the thin films have considerably fewer number of edges, therefore the intensity of the longer wavelength emission is very low, and hence completely coincides within the shorter wavelength emission band. To justify this phenomenon, we compare two literature report on PL spectra of BA_2_PbI_4_, where the reported microcrystals possessed dual emission at 521 and 561 nm, and the reported thin film possess only single emission at shorter wavelength (521 nm).^[^ ^23^ ^]^ Figure 3d and Figure S10 (Supporting Information) presents the top‐view SEM image of the recrystallized film, from which we can see a well coverage and pinhole‐free surface, enabling a better charge injection with less losses. Similarly, thin films of 4FPSI were prepared using a conventional method. This involved dissolving commercially available SnI_2_ and 4‐fluorophenethylammonium iodide in a mixture of DMF and DMSO (4/1 V/V). The rest of the procedure for thin film deposition remained unchanged, including the injecting contacts and device architecture, and this sample is referred to as the reference sample. The characterizations of the reference thin films, including absorbance, PL, XRD pattern, and SEM image, are presented in Figure S11a–c (Supporting Information). Notably, the reference thin films exhibit a comparable XRD pattern and optical characteristics to the two‐step recrystallized 4FPSI thin films. The photoluminescence quantum yield (PLQY) of the two‐step recrystallized film was determined to be 3.1%, which represents a significant improvement over the reference film, which exhibited a PLQY of ≈1.2%. This enhancement in PLQY can be attributed to the improved charge recombination dynamics, likely resulting from a reduction in defect sites achieved through our modified recrystallization methodology. Corresponding to the PLQY analysis, the TRPL studies provide enhanced carrier lifetime value for the two‐step recrystallized 4FPSI film (7.01 ns) over the reference film (6.71 ns), see Figure S12 (Supporting Information). Table S1 (Supporting Information) shows the PLQY and TRPL data comparison between the sample and reference films.

We have also performed X‐ray photoelectron spectroscopy (XPS) analysis to emphasize the role of acetic acid to maintain the stability of the microcrystals, see Figure S13 (Supporting Information). Importantly, acetic acid acts as a ligand that effectively shields the microcrystals, thereby providing enhanced stability. This prediction has been justified with the XPS analysis to investigate the surface chemistry of the synthesized microcrystals. The Sn 3d_5/2_ XPS spectrum reveals that the Sn^2^⁺ peak for the microcrystals is centered at 486.4 eV, whereas the reference sample exhibits the peak at 486.2 eV. This 0.2 eV shift is indicative of the interaction between Sn^2^⁺ ions and acetate ions on the microcrystal surface. The shift suggests a stronger bonding state of Sn^2^⁺ in the microcrystals compared to the reference sample, highlighting a significant interaction between CH₃COO⁻ and Sn^2^⁺ at the surface. Additionally, the I 3d_5/2_ XPS spectrum provides evidence for anticipated hydrogen bonding interactions. In the microcrystals, the I 3d_5/2_ peak is observed at a binding energy ≈0.2 eV lower than that in the reference sample. This shift to a lower binding energy can be attributed to the perturbation of the iodide electronic cloud due to hydrogen bonding interactions between the proton of acetic acid and the iodide ions from the perovskite structure. This hydrogen bonding likely stabilizes the microcrystals and contributes to the overall enhanced stability and performance of the synthesized material. The comparison of the Sn 3d and I 3d XPS spectra between the reference and the target thin films are provided in Figure S14 (Supporting Information). Further, an outstanding performance has been recorded for the microcrystals thin film, where Sn^2+^/Sn^4+^ is 3.1, while the reference thin film is highly oxidized, and the value is 0.1, see Table S2 (Supporting Information). The mitigation of Sn^2+^ oxidation is achieved due to the act of acetic acid ligand, which inevitably protects from moisture and aerobic oxidation. To further provide the insight of hydrophobicity over the 4FPSI thin films, we have conducted the contact angle experiment, and compared with the reference thin film, see Figure S15 (Supporting Information). The two‐step recrystallized 4FPSI thin film exhibits a contact angle of 49.1°, which is significantly higher than the 41.3° contact angle observed in the reference sample. This increased contact angle indicates a greater degree of hydrophobicity in the MC‐4FPSI thin film. The acetic acid ligands likely form a protective hydrophobic layer on the surface, reducing the affinity for water and thereby increasing the contact angle.

Following our approach, a variety of mixed halide (bromide and iodide) perovskites can be synthesized by mixing HBr along with HI during the synthesis. Further, this material was recrystallized in the form of thin film following the same approach explained before. The XRD patterns of mixed halide at different I‐to‐Br ratio is provided in Figure S16a (Supporting Information), showing identical pattern influenced from the spacer cation layer stacking with the inorganic layers. With the increase of Br‐content, they have a hypsochromic shift in absorption and PL due to the widening of bandgap with gradual Br‐mixing, shown in Figure S16b,c (Supporting Information). However, the relative PL intensity was decreased with the increase of Br content. The same synthesis strategy is followed for the preparation of FASnI_3_ powder and recrystallized for the preparation of the thin film. Figure S17a (Supporting Information) provides the XRD patterns of as prepared FASnI_3_ microcrystal powder, and FASnI_3_ thin film using these powders as precursor. In this regard, an attempt to the synthesis of quasi‐2D tin iodide perovskite is synthesized introducing both small, formamidinium (FA^+^), and bulky, 4FPEA, cations, which formed mixed phase of (4FPEA)_2_SnI_4_ (n = 1) and (4FPEA)_2_FASn_2_I_7_ (n = 2), the measured XRD patterns of the microcrystal powder and the corresponding prepared thin film is provided in Figure S17b (Supporting Information). The optical absorbance spectrum from the thin film is provided in Figure S18a (Supporting Information), where the peaks from n = 1 and n = 2 are centered at 615 and 675 nm, respectively and the corresponding PL spectra from the microcrystal powder and from the thin film is provided in Figure S18b,c (Supporting Information). Tailoring of FA^+^ and 4FPEA ratio controlled the mixed phase formation of quasi‐2D tin iodide perovskites.

**Figure 4:**
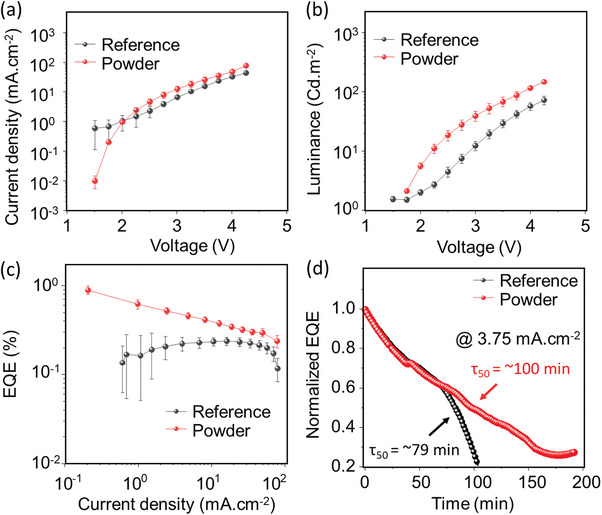
4FPSI PeLED characterizations. Plots of a) Current density‐voltage, b) luminance‐voltage, c) EQE‐current density, and d) normalized EQE‐operational time at continuous working conditions with a current density of 3.75 mA.cm^−2^.

Beyond the excellence of our synthesis protocol to provide the low‐cost preparation and the stability of as prepared microcrystal powder able to be used as precursor for thin film deposition, we studied the application of these thin films for the fabrication PeLED devices. PeLED devices were fabricated, and their performances were tallied with the traditional active layer thin film preparation, using SnI_2_ and 4‐fluorophenethylammonium iodide precursors, denoted as reference PeLED. The PeLED devices were fabricated with the following device architecture:^[^ ^24^ ^]^ ITO/PEDOT:PSS/4FPSI/PO‐T2T/LiF/Al (see Supporting Information for more details). The comparison between the reference and two‐step recrystallized powders is shown in **Figure** **4**. Figure 4a provides the current density‐voltage characteristics of the PeLED devices showing higher current densities for devices prepared from microcrystal powder precursors. It can be observed in Figure 4 that devices made of with the microcrystal powder synthesized with the new methodology showed improved luminance, see Figure 4b, EQE, see Figure 4c, and stability which has been obtained comparing with the half‐life time (τ_50_), see Figure 4d. τ_50_ is defined as the time in which initial luminance drops to half value, under continuous working conditions, in this case a current 3.75 mA, which corresponds to a luminance of 10 and 6 Cd^.^m^−2^ for powder‐based and reference samples, respectively. The powder‐based device cradles a τ_50_ of 100 min compared to 79 min for the reference sample, which rapidly dies after τ_50_, while the powder‐based LED still conserves 30% of the initial EQE even after 200 min. The PeLED devices made from the powder samples showed a maximum luminance of 166.1 cd**^.^** m^−2^ at an applied voltage of 4.25 V, which is considerably improved in comparison to the reference sample (117.7 cd**^.^** m^−2^). Likewise, an average EQE of 0.9% (maximum 1.0%) is recorded for the powder‐based samples, while the reference sample has an average EQE of 0.23% (maximum 0.3%). EQE‐voltage plot is provided in Figure S19 (Supporting Information). A list of the reported 2D tin iodide PeLED performances are provided in Table S3 (Supporting Information).

**Figure 5:**
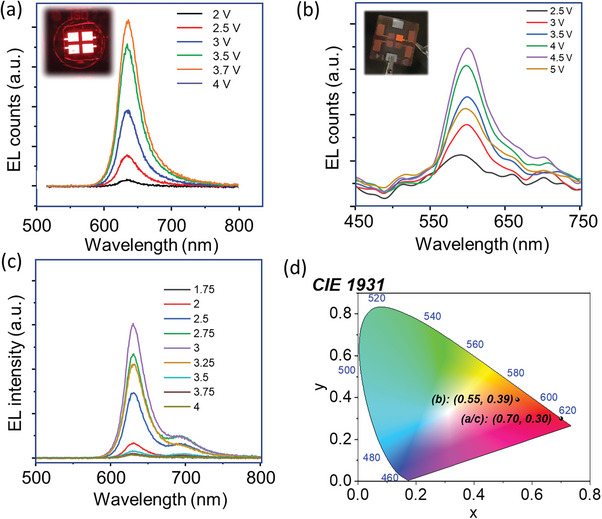
Electroluminescence spectra of PeLED devices prepared with a) (4FPEA)_2_SnI_4_, b) (4FPEA)_2_Sn(I_0.75_/Br_0.25_)_4_, and c) (4FPEA)_2_SnI_4_/(4FPEA)_2_FASn_2_I_7_. d) CIE color chromaticity diagram of all the three EL spectra presented in (a–c). Noteworthy, the positions of (a,c) are at the same coordinate.

The EL spectra of the 4FPSI based LEDs at different applied voltages is shown in **Figure** **5**a where the EL peak is centered at 634 nm, with the FWHM of 40 nm. Note that this wavelength is the optimum for red color in Rec 2020 screen standards.

As the hypsochromic shift in PL emission was observed due to Br‐alloying, an orange emitting LED device from the (4FPEA)_2_Sn(I_0.75_/Br_0.25_)_4_ powder sample has been prepared. Figure S20 (Supporting Information) represents the PeLED device performance from (4FPEA)_2_Sn(I_0.75_/Br_0.25_)_4_ as an active layer. Figure S20a,b (Supporting Information) provides the current density‐voltage and the luminance‐voltage plot, where a maximum luminance of 6 cd m^−2^ is recorded at 4.5 V. Figure S20c (Supporting Information) shows the EQE‐voltage plot, where maximum EQE of ≈0.05% is recorded. Figure 5b shows a set of EL spectra at different applied voltages. The inset of Figure 5b shows illuminated pixel from this device at 4 V. As was the case for the PL emission decrease in course of successive Br‐alloying, the obtained EL was also quite poor in this case.

To expand the scope of our material processing methodology, we also carried out mixed cation alloying by introducing formamidinium cation (FA^+^) to prepare quasi‐2D perovskites with higher order of “n”. An attempt to such preparation leads to the formation of mixed phase of n = 1 and n = 2, and their as‐prepared LED devices show an existence of dual peak from the said phases. PeLED devices were fabricated with this material following the same device architecture as done for 4FPSI, and the device characterizations are provided in Figure S21 (Supporting Information). Figure S21a,b (Supporting Information) provides the current density‐voltage and the luminance‐voltage characteristics. Corresponding EQE‐voltage graph is presented in Figure S21c (Supporting Information), and the set of EL spectra is presented in Figure 5c. Notably, while the FA‐incorporation influences on the existence of dual EL peak originated from the n = 1 (634 nm) and n = 2 (700 nm) phases, see Figure 5c, we obtained the highest luminance of this study, 270 cd m^−2^, It is one of the highest reported in the literature.^[^ ^10^ ^]^ Figure 5d displays the CIE chromaticity coordinates of the EL spectra, see Figure 5a–c. Remarkably, the red EL, originating from quasi‐2D perovskite, shares the same coordinate value as 4FSPI PeLEDs, corresponding to pure red color.

Apart from the synthesis of 2D perovskites, the NIR LED was fabricated with FASnI_3_ microcrystal powders. Figure S22 (Supporting Information) provides a set of EL spectra at different applied voltages. While this preliminary results from FASnI_3_ perovskite powder produced using the methodology reported here is below the recently reported promising FASnI_3_ NIR PeLED,^[^ ^25^ ^]^ however, this result endorses the proof of concept of wide applicability and suitability of our developed synthesis protocol, and further research following this approach to synthesize FASnI_3_ and related 3D tin halide perovskites might be interesting for future research.

## 3 Conclusion

We have developed a low‐cost synthesis strategy for the fabrication of high‐quality Sn based halide perovskite microcrystals as a competitive alternative to traditional synthesis method for both 3D and 2D tin perovskites. Addressing key challenges related to stability, precursor cost, and PeLED device performance and stability, the developed synthesis strategy offers a robust and cost‐effective approach. In this developed methodology, the choice of solvent played the key role to synthesize high‐quality, ultra‐stable, and scalable product. Acetic acid helped to enhance the product quality and scalability by keeping water away during the synthesis, and the stability governed by the creation of acetic acid mediated hydrophobic network over the perovskite, which helped to purify the microcrystals and the resulting microcrystal powder exhibits impressive ambient stability and long‐term inert atmosphere storage capabilities. Further, we have fabricated PeLEDs emitting from orange to NIR, including pure red LED with emission at 634 nm, alongside the possibility of fabricating orange‐tuned emission with halide mixing (iodide/bromide), which has been reported for the first time. Moreover, the two‐step recrystallization process for thin film fabrication yields PeLED devices with enhanced performance and operational stability compared to the reference devices using traditional methods, which endorse the product quality following our developed synthesis strategy. Enhanced performance can be mostly attributed to a higher stability originated by the lower amount of oxidized Sn^4+^ in the devices fabricated by the two‐step recrystallization process. This work not only contributes to the advancement of tin iodide perovskites but also establishes a versatile generic synthesis methodology for the preparation of various hybrid tin perovskites microcrystal powders, opening new possibilities for optoelectronic applications.

## Conflict of Interest

The authors declare no conflict of interest.

## Supporting information

Supporting Information

## Data Availability

The data that support the findings of this study are available from the corresponding author upon reasonable request.
