# Supplementary material for: Synthesis of Hybrid Tin‐Based Perovskite Microcrystals for LED Applications
Source: Adv Sci (Weinh). 2024 Jul 8;11(34):2403835. doi: 10.1002/advs.202403835 (PMC11425840; doi:10.1002/advs.202403835)
Supplement: Supplementary file 1 — Supporting Information [file ADVS-11-2403835-s001.pdf]

ADVANCED SCIENCE
Open Access

Supporting Information

for *Adv. Sci.*, DOI 10.1002/advs.202403835

Synthesis of Hybrid Tin-Based Perovskite Microcrystals for LED Applications

Jesus Sanchez-Diaz, Jhonatan Rodriguez-Pereira, Samrat Das Adhikari and Iván Mora-Seró**

Supporting Information

Synthesis of hybrid tin iodide perovskite microcrystals and their LED applications

Jesus Sanchez-Diaz, Jhonatan Rodriguez-Pereira[2,3], Samrat Das Adhikari,* and Iván Mora-Seró*

[1]Institute of Advanced Materials (INAM), Universitat Jaume I. Av. de Vicent Sos Baynat, s/n 12006, Castelló de la Plana, Spain.

[2]Center of Materials and Nanotechnologies, Faculty of Chemical Technology, University of Pardubice, Nam. Cs. Legii 565, 53002 Pardubice, Czech Republic.

[3]Central European Institute of Technology, Brno University of Technology, Purkynova 123, Brno, 612 00, Czech Republic

Experimental section.

Materials: Acetic acid (AcOH, 99-100 %, sigma aldrich), hydroiodic acid (HI; 57 wt. % in H_2O, distilled, stabilized, 99.95 %), hydrobromic acid (HBr; 48 wt. % in H_2O), hypophosphorus acid (H_3PO_2; 50wt. % in H_2O), Tin(II) iodide (SnI_2, 99.99%), tin(II) fluoride (SnF_2, 99%), N,N-dimethylformamide (DMF, 99.8%), and dimethylsulfoxide (DMSO, 99.8%) were purchased from Sigma-Aldrich. 2,4,6-tris[3-(diphenylphosphinyl)phenyl]-1,3,5-triazine (PO-T2T) was purchased from Lumtec Taiwan. 4-Fluoro-Phenethylammonium iodide (4F-PEAI. 98%) was purchased from Greatcell solar materials. PEDOT: PSS 4083 aqueous solution was purchased from Heraeus. Tin (II) oxide (SnO; 99%) was purchased from Alfa Aesar, 4-fluorophenethylamine (4FPEA;) was purchased from TCI. All materials were use as received with no further purifications.

Synthesis of 4FPSI microcrystals powder: A mixture containing 3 ml of acetic acid, 0.2 ml of HI, and 0.05 ml of H_3PO_2 was combined in a 3-necked flask under an inert atmosphere of nitrogen (N_2). The mixture was then treated at room temperature for 30 minutes. The presence of a faint red to colorless appearance confirmed the stabilization of HI in the presence of H_3PO_2. Next, 134 mg of SnO was introduced into the flask, and the temperature was raised to 100 °C. The solution was treated for an additional 30 minutes until it displayed an orange color. Subsequently, 0.26 ml of 4FPEA was added to the solution, and the reaction temperature was raised to 135 °C. The mixture was treated for 15 minutes under these conditions. After completion of the reaction, the solution was cooled using an ice bath for approximately 2 minutes. The product was then collected by vacuum suction filtration. To remove excess iodine from the product, the collected powder was washed with hexane three times using vacuum suction filtration. Finally, the powder was dried. The dried powder was stored inside the glovebox. The product yield was 320 mg. The scale up synthesis of 4FPSI was carried out by taking every reagents and solvent twenty times, and the product yield was 6.5 g, which is also twenty times of the batch synthesis, see **Figure S8c**.

Synthesis of PSI microcrystals powder: PSI was synthesized following the same protocol of 4FPSI by replacing 4-fluorophenethylamine in place of phenethylamine.

Synthesis of $(4FPEA)_2Sn(I/Br)_4$ powder: The same methodology as for 4FPSI was followed, where a mixture of HI and HBr was added with altering their respective proportions.

Synthesis of quasi 2D $(4FPEA)_2SnI_4/(4FPEA)_2FASn_2I_7$: 3 ml of acetic acid, 0.2 ml of HI, and 0.05 ml of H_3PO_2 were loaded together in a 3-necked flask under inert atmosphere, and treated for 30 minutes following the above-mentioned procedures. In the next step, 1 mmol of SnO was introduced, and the temperature was raised to 100 °C, and treated for 20-30 minutes. In the next step, calculated amount (2/n*1 mmol) of 4-fluorophenethylamine (0.13 ml for n=2) was added into the solution, followed by the addition of calculated amount ((n-1)/n*1 mmol) of formamidinium acetate (52 mg for n=2), while keeping at 100 °C and treating it for 15 minutes. The product was collected by vacuum suction filtration

under hot condition (~70-100 °C). The powder was washed with hexane for 3 times to remove excess iodine from the product. Noteworthy, the calculation for this preparation was made for n=2, but the synthesized product was a mixture of n=1 ($(4FPEA)_2SnI_4$) and n=2($(4FPEA)_2FASn_2I_7$).

Synthesis of $FASnI_3$ microcrystal powder: In a 3-necked flask under an inert atmosphere, a mixture consisting of 3 ml of acetic acid, 0.2 ml of HI, and 0.05 ml of H_3PO_2 was treated for 30 minutes following the mentioned procedure. In the next step, 134 mg of SnO was added to the flask, and the temperature was raised to 100 °C. The mixture was treated for an additional 30 minutes. Once the solution displayed an orange color, 104 mg of formamidinium acetate ($FASnI_3$) was introduced, causing an instantaneous change in the color of the precipitate powder from orange to black. This color change confirmed the formation of $FASnI_3$ perovskite microcrystals. The reaction was allowed to continue for 2-3 minutes at 100 °C to complete the process. The solid powder was collected and stored inside the glovebox.

The hot solution was then filtered using vacuum suction to collect the product. The obtained powder was subjected to three washes with hexane, followed by vacuum suction filtration, to remove excess iodine from the product.

Colloidal synthesis of PSI microcrystals: In a 10 ml vial, 0.135 gm of SnI_2 were dissolved in 1.5 ml of trioctylphosphene (TOP) and maintained under an inert atmosphere. Simultaneously, in a 3-necked flask, a mixture of 0.5 ml of oleic acid and 5 ml of 1-octadecene was combined and degassed with nitrogen for 30 minutes at 120 ^oC. Subsequently, the SnI_2-TOP solution was injected into the reaction vial. Following this, 0.4 ml of phenethylamine was injected into the reaction flask at 120 ^oC and allowed to react for 5 minutes. Finally, the resulting powder was collected by centrifugation, washed three times with hexane, and the final precipitate was redispersed in hexane.

Precursor solution preparation

Powder-based solution: 0.1 M of 4-$FPEASnI_4$ microcrystals and 0.01 M of SnF_2 were dissolved in a solvent mixture of DMF:DMSO (4:1) and stirred at room temperature over night.

Reference solution: 0.16 M precursor solution was prepared by dissolving stoichiometric 4F-PEAI and SnI_2 in a solvent mixture of DMF:DMSO (4:1) and stirred at room temperature over night, 0.016M of SnF_2 was added to the precursor solution.

Device Fabrication

ITO substrates were clean with Soap water, Ethanol, acetone and isopropanol, respectively in a ultrasonic bath for 15 min each, after the cleaning procedure, they were dry with N_2. Before the hole transporting layer (HTL) deposition, the substrates were treated with UV-Ozone for 20 minutes. We used PEDOT:PSS as HTL and the solution was prepared as follow; 5 mg/ml of Nicotinamide (Nico) was added to PEDOT:PSS, in order to adjust the work function and have a better alignment with our Sn-Perovskite layer.[1] The Nico-PEDOT:PSS solution was sonicated for 5 minutes and the filtered with a 0.45 µm PVDF filter and spin-coated at 3500 rpm for 40s and annealed for 130 ^{o}C for 20 min. After Nico-PEDOT:PSS deposition, the samples were transferred to a N_2 filled glovebox for the perovskite film deposition. The perovskite solution (Microcrystals or Reference) was filtered with a 0.22 µm PTFE filter, then it was deposited by spin-coating at 4000 rpm and 800 of acceleration for 50s, afterwards a two-step annealing was performed at 65 ^{o}C and 90 ^{o}C for 1 minute and 12 minutes, respectively. Finally, the substrates were transferred to a vacuum chamber, to evaporate 40 nm of PO-T2T, 1 nm of LiF and 100 nm of Al. The area of the device was defined by the evaporation mask, which was 0.08 cm^2.

Characterization

- SEM images were taken with a field emission scanning electron microscope (FEG-SEM) JEOL 3100F) operated at 15 kV.
- XRD pattern of the films were measured using X-ray diffractometer (D8 Advance, Bruker-AXS) (Cu Kα, wavelength λ=1.5406 Å) with a Bragg angle range of 4-70° and step size of 0.05°.
- Absorption spectra were registered on a Varian 20Cary300BIO UV/VIS spectrophotometer.

- Surface chemical composition and electronic state of Tin Iodide MC samples were determined by X-ray Photoelectron Spectroscopy (XPS, ESCA-2SR, Scienta-Omicron). Spectra were recorded using monochromatic Al Kα = 1486.6 eV, operated at 100W. The charge was controlled with the charge neutralizer (CN-10) operated at 5μA and 1.2eV. The survey and high-resolution spectra were recorded at a pass energy of 150 and 30 eV, respectively. Binding energy scale was referenced to adventitious carbon (284.8 eV). CasaXPS processing software (Casa software Ltd) was used to analyze the data and the quantitative analysis was made using sensitivity factors provided by the manufacturer. The LED characterization (EL spectra, J–V–L curves, luminance, and EQE) was done by using a Hamamatsu EQE measurement system (C9920-12) coupled with an integrating sphere connected to a PMA-12 photonic multichannel detector and a Keithley 2400 instrument as a current/voltage source meter.

Calculation of d-spacing from Bragg's equation:

Bragg's equation[2]: $n\lambda = 2d \sin\theta$; where λ is wavelength, θ is incident angle, n is integer. Here, n = 1; λ is 1.5406 A°, and θ is (5.39/2 = 2.195). Hence, d = 1.64 nm.

Supporting figures

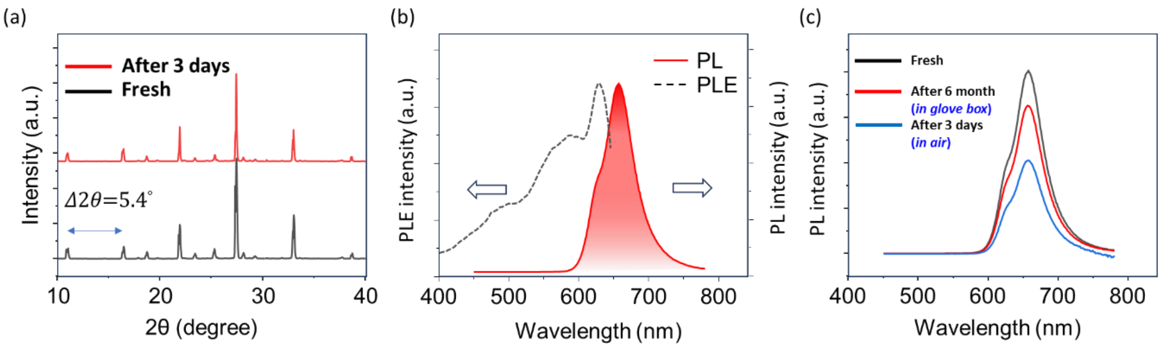

Figure S1. Characterizations of PSI perovskite microcrystals. (a) XRD pattern of freshly prepared and after 3 days of ambient storage. (b) PL/PLE spectra of the freshly prepared sample. (c) A comparison of PL spectra of the PSI powders between the fresh, after 6 months stored inside the glove box, and 3

days in ambient atmosphere. The PL was recorded at 405 nm excitation. The PLE was recorded at 650 nm.

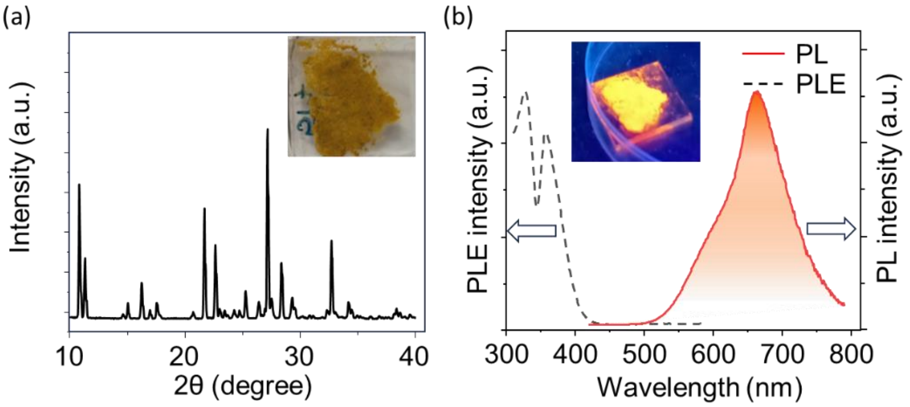

Figure S2. Characterization of degraded PSI perovskite microcrystals synthesized following traditional method.[3] (a) XRD pattern and the inset shows the degraded perovskite turned yellowish, which was black before degradation. (b) PL/PLE of degraded perovskite, where the degraded perovskite has the phase transformed product as a byproduct provides STE emission. Noteworthy, the phase-transformed product is not exclusive.

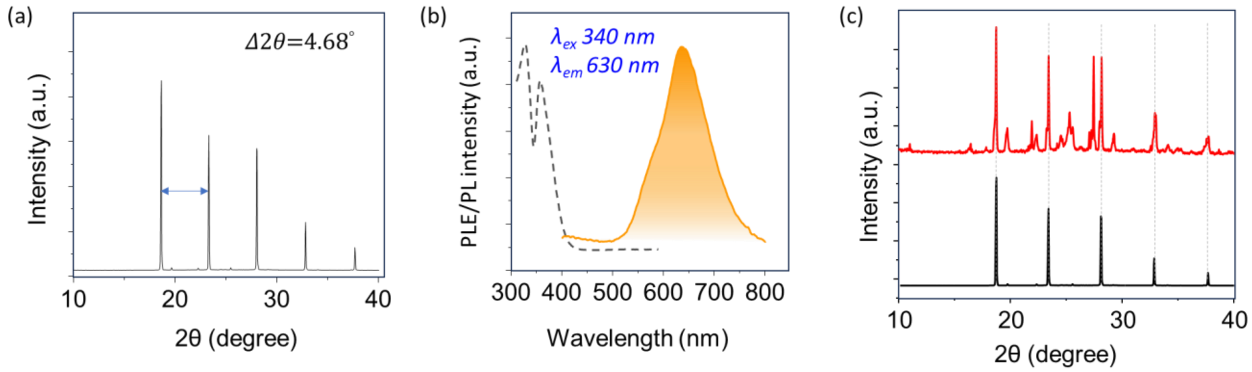

Figure S3. (a) XRD pattern, and (b) PL and PLE spectra of colloidally synthesized PSI microcrystals. (c) A comparison of the XRD patterns from Figure S2a and Figure S3a to show the phase transformation.

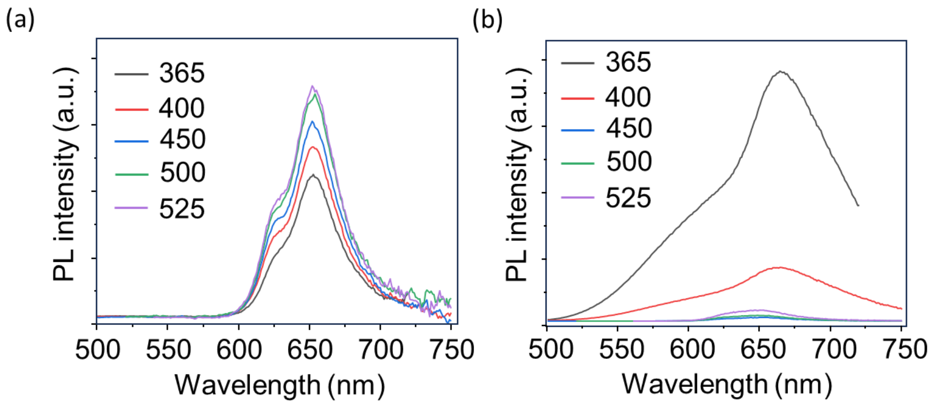

Figure S4. PL spectra of (a) 4FPSI, and (b) PSI recorded at varied excitation wavelengths. These data evidence the existence of STE emission in PSI because of phase transformation, while 4FPSI provides only free excitonic emission. Herein, PSI and 4FPSI has been synthesized following the traditional approach (not using acetic acid as solvent).

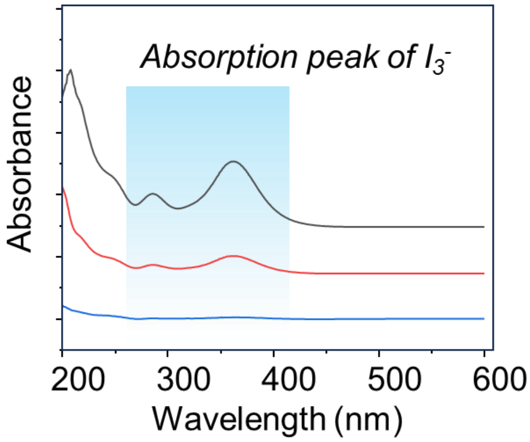

Figure S5. UV-vis absorption spectra of supernatant hexane collected from the successive washing of PSI sample.

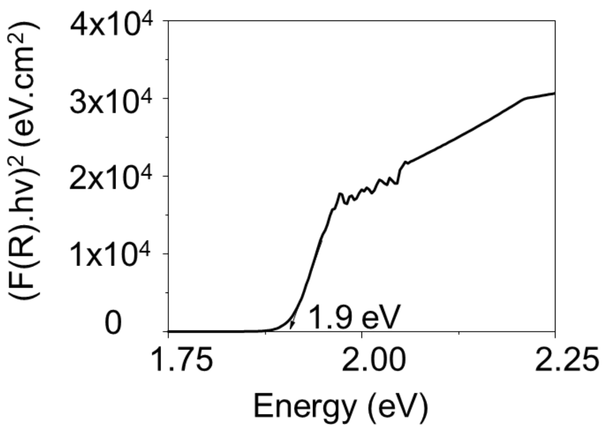

Figure S6. Tauc plot of 4FPSI microcrystals perovskite extracted from the reflectance spectrum presented in Figure 2d.

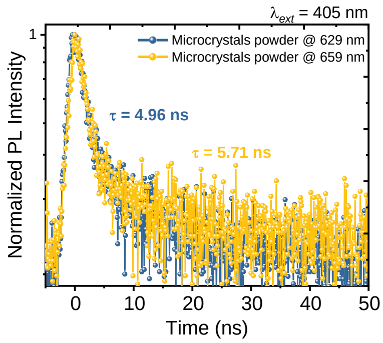

Figure S7. TRPL spectra of 4FPSI microcrystals at the emission wavelength of 629 nm (blue plot), and 659 nm (yellow plot).

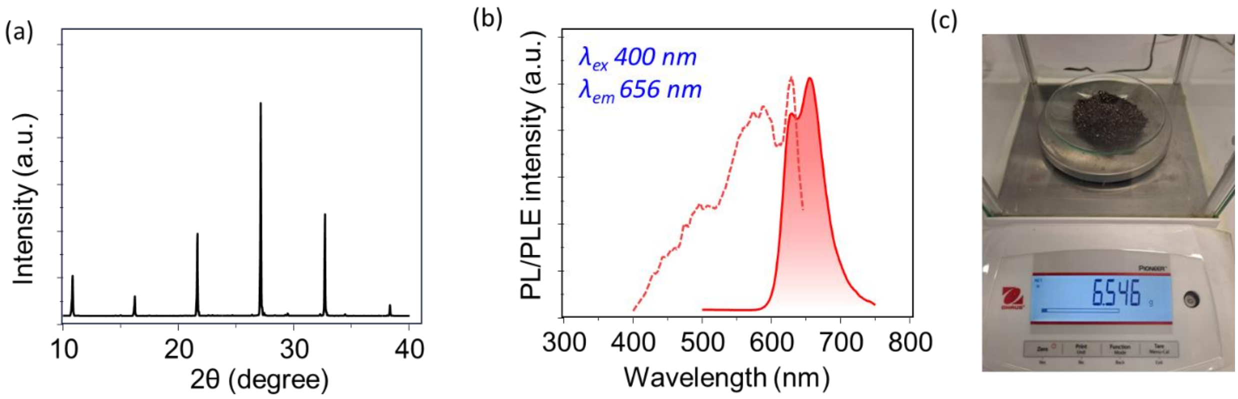

Figure S8. Characterizations of twenty-time scale-up 4FPSI microcrystals: (a) XRD pattern, and (b) PL and PLE spectra. (c) A photograph of the scale-up product yield (details are provided in experimental section). Results are similar with low-scale synthesis reported in **Figure 2.**

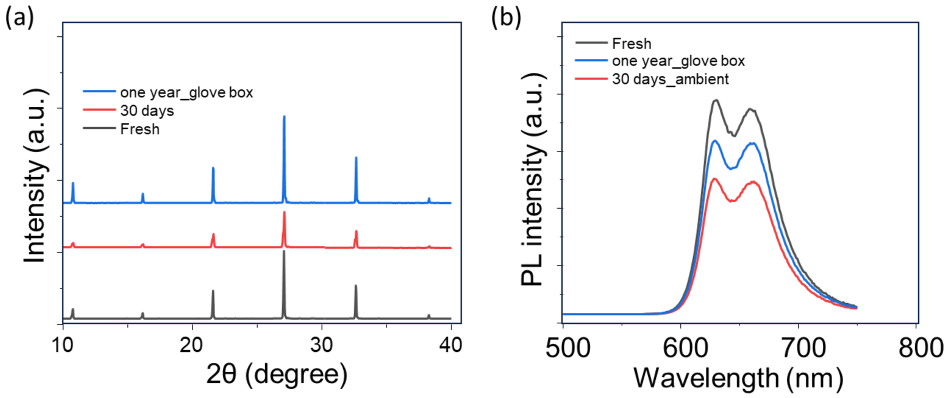

Figure S9. (a) XRD patterns and (b) PL spectra of freshly prepared (black line), 30 days storage in ambient (red line), and one year storage inside the glove box (blue line).

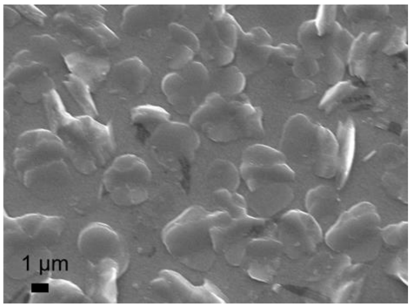

Figure S10. Top-view SEM image of two-step recrystallized 4FPSI thin film at different resolution.

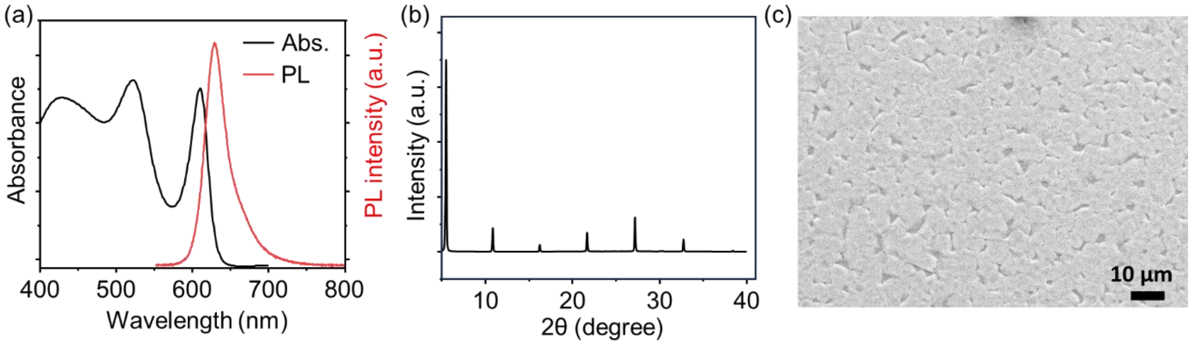

Figure S11. (a) UV-vis absorption and PL, (b) XRD pattern, and (c) SEM image of 4FPSI reference thin film.

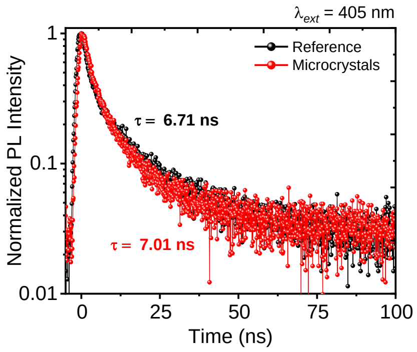

Figure S12. TRPL spectra of the thin films: microcrystal thin film (red plot) and reference thin film (black plot).

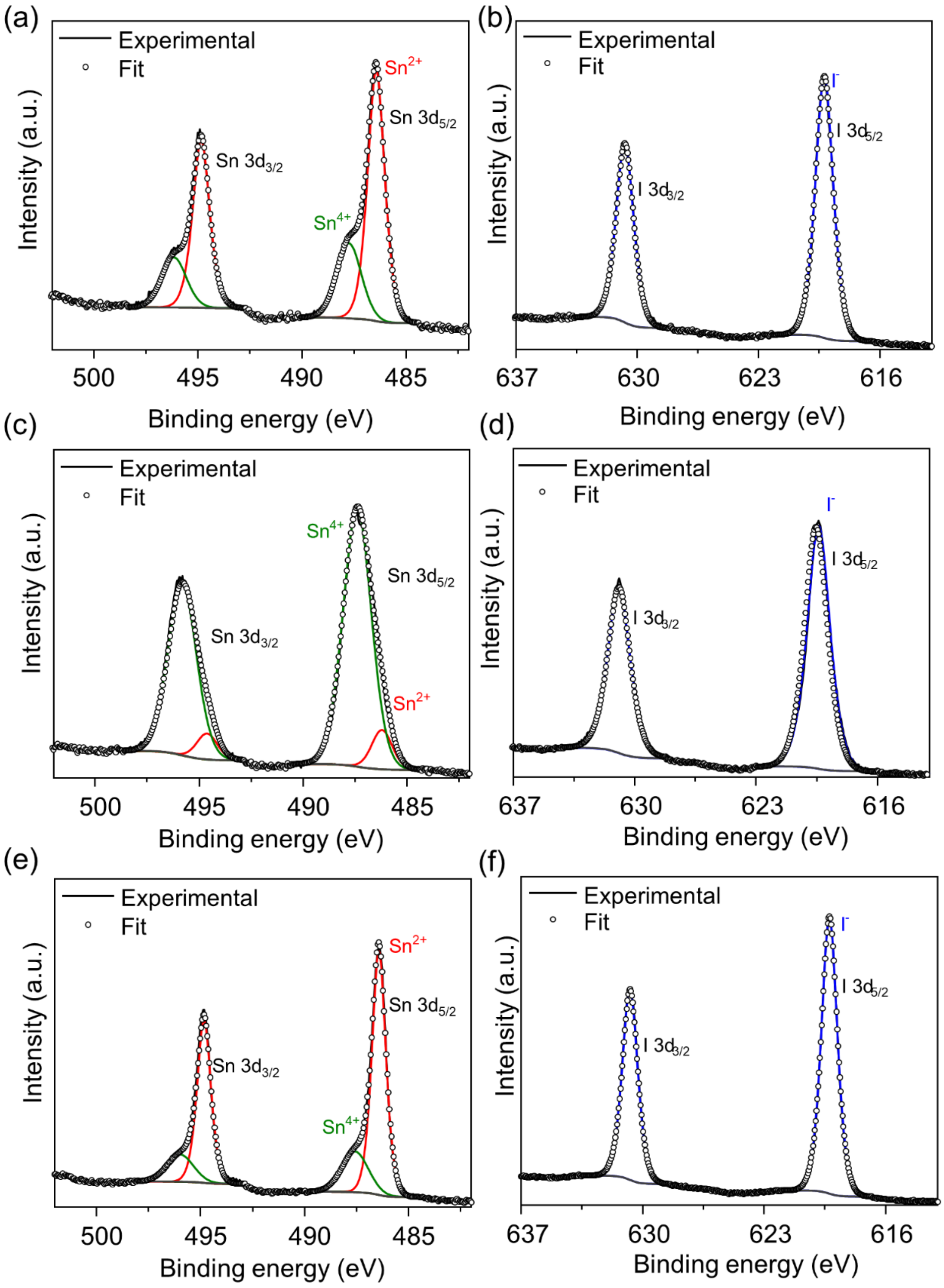

Figure S13. XPS spectra of (a-b) 4FPSI microcrystal powder, (c-d) reference thin film, and (e-f) microcrystal recrystallized thin film.

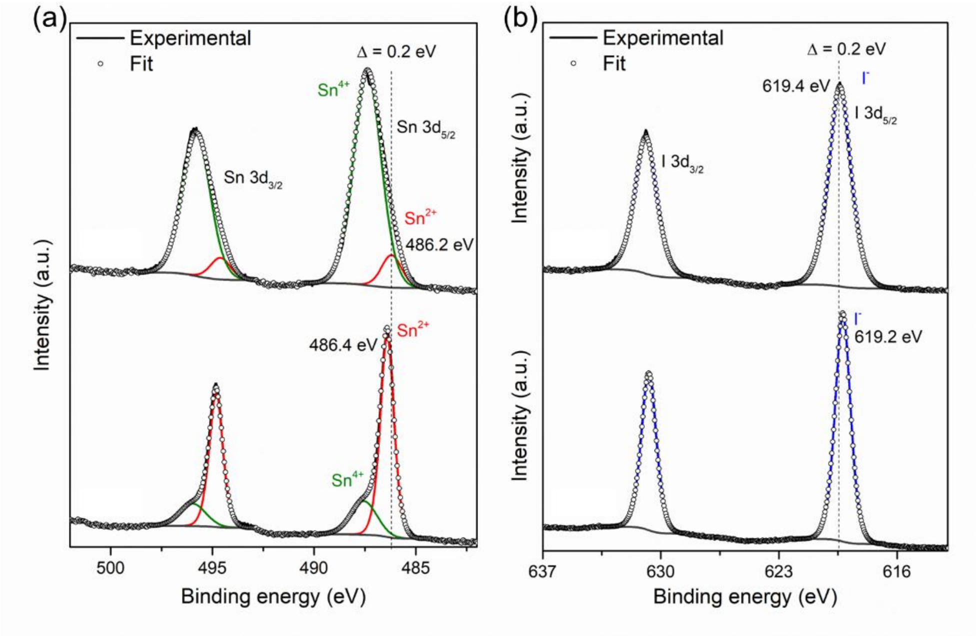

Figure S14. Comparison of (a) Sn-3d and (b) I-3d XPS spectra between the reference (upper panel) and the microcrystal recrystallized thin films (bottom panel).

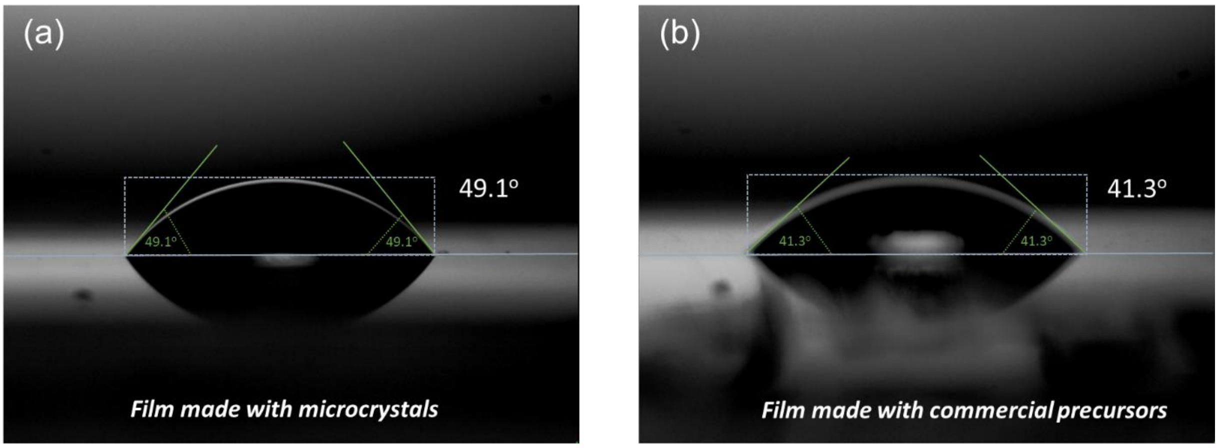

Figure S15. Contact angle measured 1 second after water touches the perovskite film made with (a) microcrystals and (b) commercial precursors.

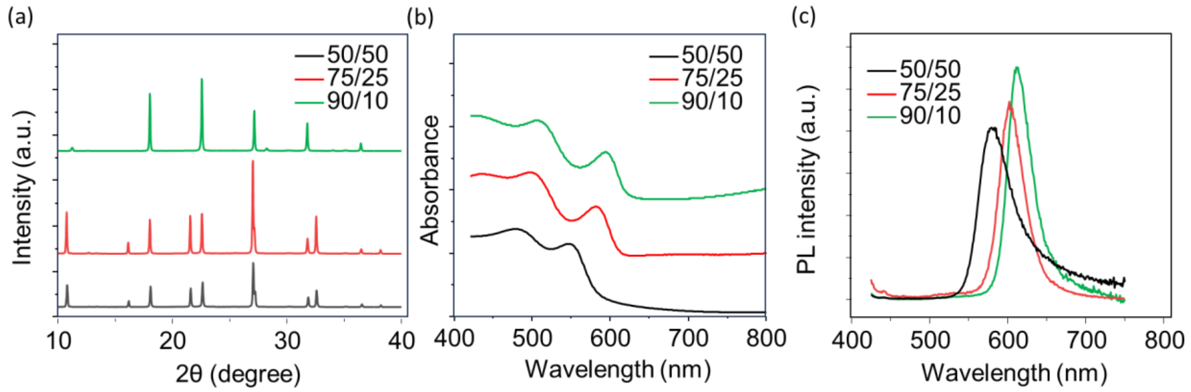

Figure S16. Characterizations of $(4FPEA)_2Sn(I/Br)_4$ with different nominal I/Br ratios, (a) XRD patterns, (b) absorbance and (c) PL spectra at 50/50, 75/25, and 90/10 I-to-Br ratio.

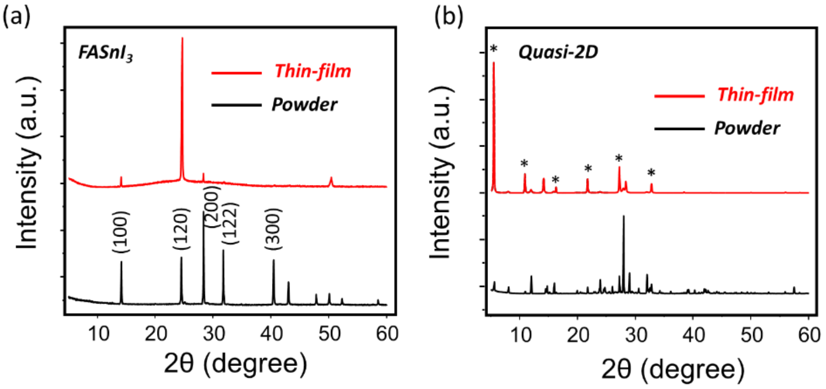

Figure S17. XRD patterns of as-prepared microcrystals and thin-films of (a) 3D-$FASnI_3$ perovskite, and (b) quasi-2D tin iodide perovskite. The asterisks in the upper panel of **Figure S6b** are assigned to n=1 phase, and the low-intense peaks corresponds to the higher ‘n’-phase.

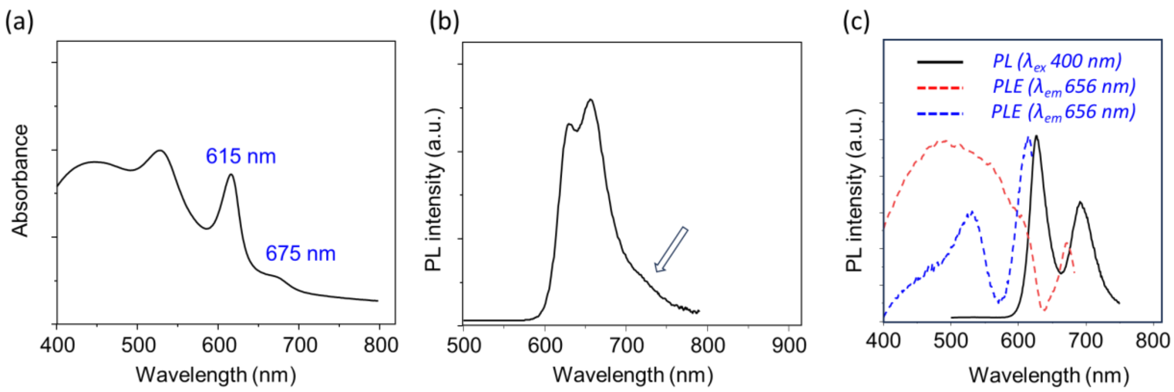

Figure S18. Optical characterization of quasi-2D tin iodide perovskite (mixed phase of $(4FPEA)_2SnI_4$ (n=1) and $(4FPEA)_2FASn_2I_7$ (n=2). (a) Absorbance spectrum of recrystallized thin film. PL spectrum of (b) the pristine microcrystal powder, and (c) the recrystallized thin film.

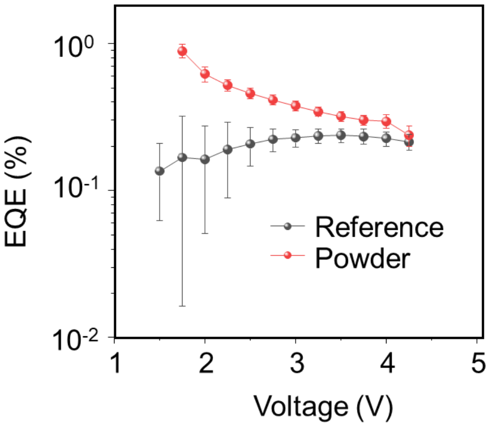

Figure S19. EQE-voltage plot of the 4FPSI LED device characterizations presented in Figure 4.

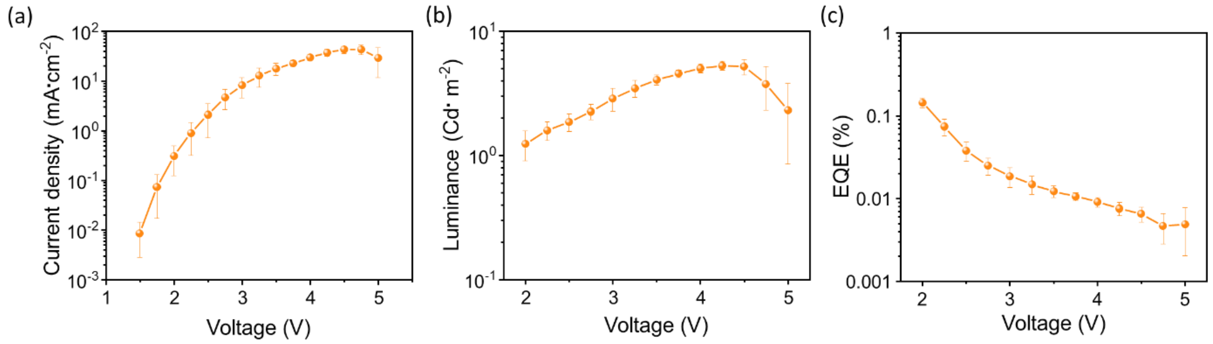

Figure S20. LED device characterizations of $(4FPEA)_2Sn(I_{0.75}/Br_{0.25})_4$ perovskites. (a) Current density-voltage, (b) Luminance-voltage, and (c) EQE-voltage plots. Corresponding EL spectra are presented in Figure 5b.

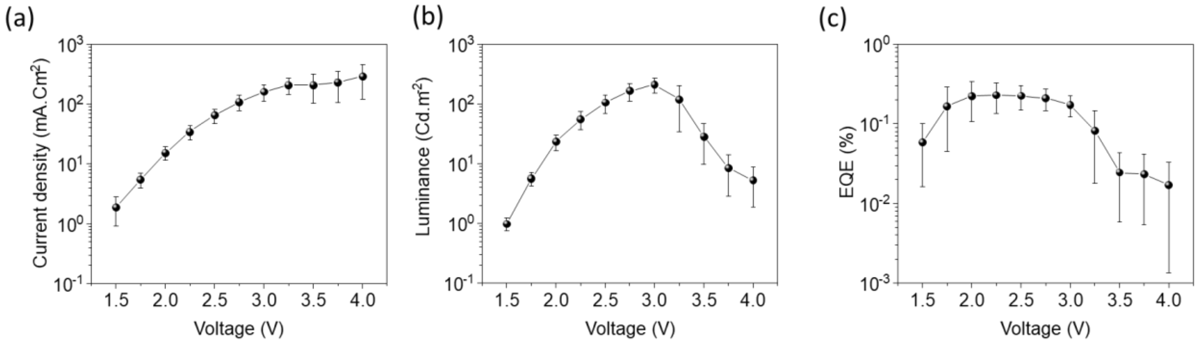

Figure S21. LED device characterizations of quasi-2D $(4FPEA)_2SnI_4/(4FPEA)_2FASn_2I_7$ perovskites. (a) Current density-voltage, (b) Luminance-voltage, and (c) EQE-voltage plots. Corresponding EL spectra are presented in Figure 5c.

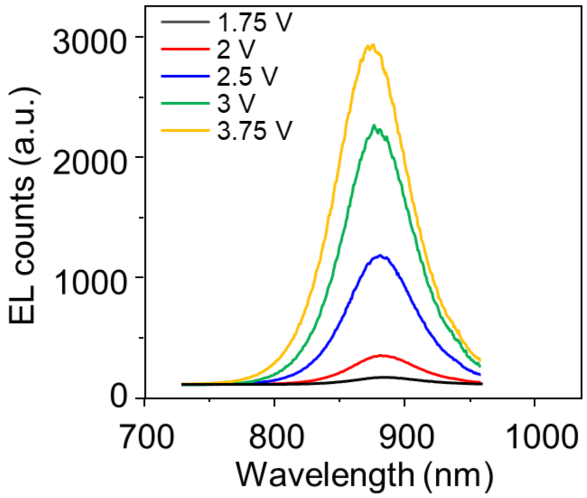

Figure S22. A set of NIR EL spectra at successive applied voltages.

Supporting tables

Table S1. TRPL and PLQY data of 4FPSI microcrystals and thin films.

Sample	PLQY (%)	Decay lifetime
4FPSI microcrystals	1.23	4.96 ns (@629 nm), 5.71 ns (@659 nm)
4FPSI (target film)	3.1	7.01 ns
4FPSI (reference film)	1.2	6.71 ns

Table S2. Component quantification analyzed from XPS spectra.

Sample Identifier	Atomic concentration [%]				Ratio
	Sn^{2+}	Sn^{4+}	I^-	Others (C, N, F..)	Sn^{2+}/Sn^{4+}
Reference thin film	0.52	4.65	11.04	83.79	0.2
Microcrystal-thin film	4.52	1.48	19.33	74.67	3.1
Microcrystal powder	3.36	1.42	15.89	79.33	2.4

Table S3. List of reported 2D tin iodide PeLEDs performances. Note that in this table is plotted the maximum reported EQE, that in some cases coincides with very low currents and electroluminescent signals. Consequently, comparison in terms of this parameter is not completely fair.

Material	Modification	*Turn-on voltage (V)*	luminance (Cd.m-2)	Maximum Reported EQE	Lifetime	Reference
PEA_2SnI_4	Pristine	*3.6*	0.15	-	-	Lanzetta et al.[4]
PEA_2SnI_4	Additive: valeric acid	-	-	5	>15 hours	Yuan et al.[5]
PEA_2SnI_4	Additive: naphthol Sulfonic Salt	*3*	132	0.72	62 sec	Gao et al.[6]
PEA_2SnI_4	Use of N,N′-dimethylpropyleneurea (DMPU) as co-solvent with DMF	-	68.84	0.36	-	Heo et al.[7]
PEA_2SnI_4	Two-step recrystallization	*2.9*	43.3	0.4	11 min	Cheng et al.[8]
PEA_2SnI_4	Additive: L-glutathione	-	328.2	9.32	-	Bai et al.[9]
TEA_2SnI_4	Pristine	*2.3*	322	0.62	~25 sec	Wang et al.[10]
TEA_2SnI_4	Additive: tautomeric mixture of cyanuric acid	*1.9*	-	20.29	27.6 hours	Han et al.[11]
$TEA2SnI_4$	Additive: Biuret	*2.5*	418	1.37	244 sec	Jia et al.[12]
4-fluorophenethylammonium tin iodide	Reference	*1.75*	117.7	0.3	79 min	This work
4-fluorophenethylammonium tin iodide	Microcrystal recrystallized	*1.7*	166.1	1	100 min	This work

References

[1] W.-F. Yang, J.-J. Cao, J. Chen, K.-L. Wang, C. Dong, Z.-K. Wang, L.-S. Liao, Solar RRL 2021, 5, 2100713.
[2] C. G. Pope, Journal of Chemical Education 1997, 74, 129.
[3] V. V. Nawale, T. Sheikh, A. Nag, The Journal of Physical Chemistry C 2020, 124, 21129.
[4] L. Lanzetta, J. M. Marin-Beloqui, I. Sanchez-Molina, D. Ding, S. A. Haque, ACS Energy Letters 2017, 2, 1662.
[5] F. Yuan, X. Zheng, A. Johnston, Y.-K. Wang, C. Zhou, Y. Dong, B. Chen, H. Chen, J. Z. Fan, G. Sharma, P. Li, Y. Gao, O. Voznyy, H.-T. Kung, Z.-H. Lu, O. M. Bakr, E. H. Sargent, Science Advances, 6, eabb0253.
[6] C. Gao, Y. Jiang, C. Sun, J. Han, T. He, Y. Huang, K. Yao, M. Han, X. Wang, Y. Wang, Y. Gao, Y. Liu, M. Yuan, H. Liang, ACS Photonics 2020, 7, 1915.
[7] Y. J. Heo, H. J. Jang, J.-H. Lee, S. B. Jo, S. Kim, D. H. Ho, S. J. Kwon, K. Kim, I. Jeon, J.-M. Myoung, J. Y. Lee, J.-W. Lee, J. H. Cho, Adv. Funct. Mater. 2021, 31, 2106974.

[8] Y.-H. Cheng, R. Moriyama, H. Ebe, K. Mizuguchi, R. Yamakado, S. Nishitsuji, T. Chiba, J. Kido, ACS Applied Materials & Interfaces 2022, 14, 22941.
[9] W. Bai, M. Liang, T. Xuan, T. Gong, L. Bian, H. Li, R.-J. Xie, Angewandte Chemie International Edition 2023, 62, e202312728.
[10] Z. Wang, F. Wang, B. Zhao, S. Qu, T. Hayat, A. Alsaedi, L. Sui, K. Yuan, J. Zhang, Z. Wei, Z. a. Tan, J. Chem. Phys. Lett. 2020, 11, 1120.
[11] D. Han, J. Wang, L. Agosta, Z. Zang, B. Zhao, L. Kong, H. Lu, I. Mosquera-Lois, V. Carnevali, J. Dong, J. Zhou, H. Ji, L. Pfeifer, S. M. Zakeeruddin, Y. Yang, B. Wu, U. Rothlisberger, X. Yang, M. Grätzel, N. Wang, Nature 2023, 622, 493.
[12] H. Jia, H. Shi, R. Yu, H. Ma, Z. Wang, C. Zou, Z. a. Tan, Small 2022, 18, 2200036.
